# Clinical profile, etiology, and outcome of hemophagocytic lymphohistiocytosis associated with histiocytic necrotizing lymphadenitis

**DOI:** 10.1007/s12519-022-00660-z

**Published:** 2023-01-03

**Authors:** Kuang-Guo Zhou, Duan-Hao Gong, Dan Peng, Zhi-Qiong Wang, Wei Huang

**Affiliations:** 1 Department of Hematology, Tongji Medical College, Tongji Hospital, Huazhong University of Science and Technology, Jiefang Road 1095#, Wuhan, 430030 China; 2 Department of Nuclear Medicine, Tongji Medical College, Tongji Hospital, Huazhong University of Science and Technology, Wuhan, China

Hemophagocytic lymphohistiocytosis (HLH) is a life-threatening clinical syndrome characterized by aberrantly activated macrophages and cytotoxic T cells, leading to multiple organ failure [1]. The main manifestations included recurrent fever, cytopenia, liver dysfunction, and sepsis-like syndrome. Physicians should be fully aware of HLH since early diagnosis and management may prevent irreversible organ failure and subsequent death. Treatment protocols, such as HLH-94 based on etoposide (VP16), are commonly used in clinical practice, especially in familial HLH [2] and malignancy-associated HLH [3].

Kikuchi-Fujimoto disease (KFD) is a rare benign disease characterized by fever and cervical lymphadenopathy. Its etiology is still unclear. KFD may also be associated with autoimmune disorders or possible viral infection [4]. Corticosteroids and/or symptomatic treatments are commonly used in KFD treatments [5]. The incidence of HLH-KFD is very rare, with only a few cases reported. Herein, we report the diagnosis and treatment of an adolescent patient with virus-induced HLH-KFD. Additionally, we analyzed the predisposing factors, treatments, and outcomes of HLH-KFD by a literature review.

A 14-year-old boy with a 2-week history of high fever was admitted to the hospital. Physical examination revealed multiple enlarged superficial lymph nodes (palpable in the bilateral neck, armpit, groin with the maximal size about 3 × 1.5 cm in the left neck) and an enlarged spleen (palpable four centimeters beneath the rib cage in the left upper abdomen). Routine blood and biochemical tests showed no abnormalities. Traditional and metagenomic next-generation sequencing (mNGS) detection of pathogenic microorganisms in the blood were negative, including Epstein-Barr virus (EBV). The autoimmune and serological tumor antigen tests revealed normality. Anti-infective treatment (penciclovir 0.25 g twice daily, linezolid 0.6 g twice daily, biapenem 0.3 g four times daily) was not interrupted after his admission. On the sixth day after admission, his fever remained along with the rapidly worsening laboratory results, which indicating a potentially fatal illness (Table 1). To select the most valuable biopsy position for identifying the etiology of persistent high fever and the extent of systemic lymphadenopathy, positron emission tomography-computed tomography (PET-CT) was performed. PET-CT showed multiple enlarged hypermetabolic lymph nodes in bilateral parapharyngeal, bilateral neck, left parotid gland area, bilateral supraclavicular area, bilateral armpit, mediastinum, retroperitoneum, bilateral iliac vessels, bilateral pelvis and bilateral groin, with the maximal value of standardized uptake value (SUVmax, 15.6) occurred in right iliac fossa and splenomegaly (SUVmax, 6.6) (Fig. 1a). Bone marrow aspiration showed significant hemophagocytosis (Fig. 1b). The patient met the diagnostic criteria of HLH. After lymph node biopsy was carried out, he immediately received treatment according to the HLH-94 protocol as VP16 (100 mg on the sixth, seventh, eleventh, twelfth day after admission; 200 mg on the 14th and 16th day) plus dexamethasone (15 mg on the sixth to the sixteenth day after admission and discontinued within 1 month) [2].

**Table 1:** Laboratory results of patient in our department after admission

Variables	Reference range	Value on the first day	Value on the sixth day	Value on the twelfth day	Value on the twentieth day
Leucocyte (× 10^9^/L)	3.50–9.50	1.64	0.96	2.01	4.09
Hemoglobin (g/L)	130–175	116	110	98	77
Platelet (× 10^9^/L)	125–350	145	145	271	132
Aspartate aminotransferase (U/L)	≤ 40	98	213	39	18
Alanine aminotransferase (U/L)	≤ 41	34	89	108	61
Lactate dehydrogenase (U/L)	120–300	1666	> 1867	517	200
Ferritin (µg/L)	30–400	10,030	22,996	1891	617.6
Granzyme B in NK cells (%)	> 78	NA	70.3	NA	NA
NK cells activity (%)	> 15.11	NA	2.2	NA	NA
CD107a in NK cells (%)	> 40	NA	22.9	NA	NA
Interleukin 1β (pg/mL)	< 5	NA	5.5	5.1	< 5
Interleukin 2 receptor (U/mL)	223–710	NA	6543	1456	924
Interleukin 6 (pg/mL)	< 7	NA	161.7	7.26	< 1.5
Interleukin 8 (pg/mL)	< 62	NA	78.8	77.8	20.3
Interleukin 10 (pg/mL)	< 9.1	NA	173	37	< 5
Tumor necrosis factor α (pg/mL)	< 8.1	NA	32.2	17.2	5.1
Creatine kinase (U/L)	≤ 190	300	395	174	NA
Fibrinogen (g/L)	2–4	3.63	3.39	2.3	2.1
D-dimer (µg/mL)	< 0.5	2.58	3.71	< 0.5	< 0.5
Fibrinogen degradation products (FDP) (µg/mL)	< 5	10.5	12.1	6.1	< 5.0

*NA* not available

**Fig. 1:**
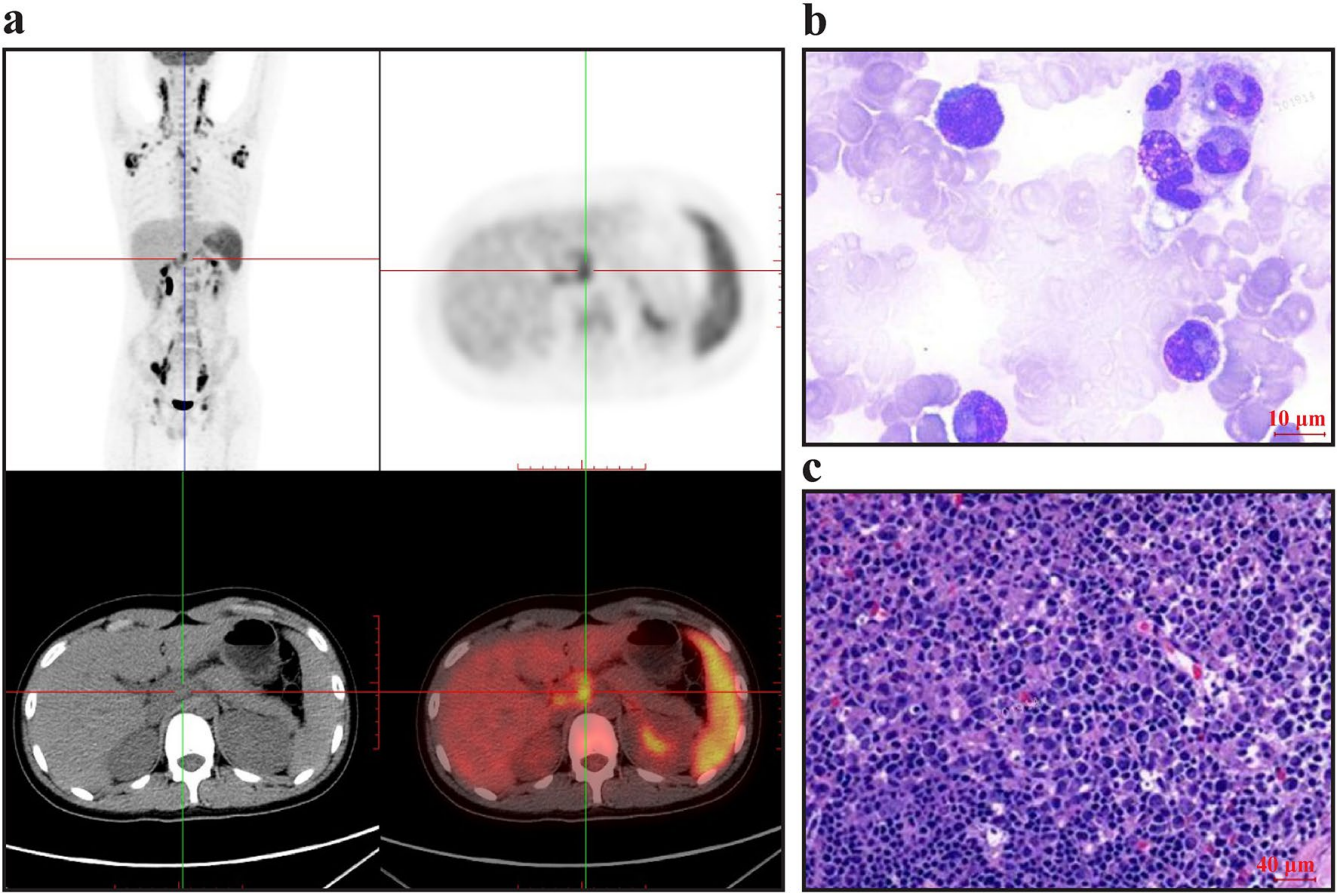
Characteristics of positron emission tomography-computed tomography (PET-CT), cytology of bone marrow, and left cervical lymph node pathology. **a** PET-CT showed hypermetabolic lymph nodes and spleen. **b** Bone marrow aspirate showed hemophagocytic histiocytes (original magnification × 1000). **c** Typical hematoxylin & eosin staining of Kikuchi-Fujimoto disease (original magnification ×40), which had histiocytes with CD163 (+), CD68 (+), MPO (scattered+), CD123 (scattered+), and lymphocytes with CD3 (+), CD7 (+), CD43 (+), CD5 (scattered+), CD8 (+), CD4 (scattered few+), GrB (+), TIA-1 (+), CD56 (scattered few +), MUM1 (scattered+), CD30 (about 15% medium intensity+), CD20 (few+), CD15 (scattered few+), CD25 (scattered few +), ALK(1A4) (–), CD35 (FDC network+), Ki67 (High, +), EBER CISH (–). *MPO* myeloperoxidase, *GrB* Granzyme B, *TIA-1* T cell intracellular antigen, *MUM1* multiple myeloma oncogene-1, *ALK* anaplastic lymphoma kinase, *EBER* Epstein–Barr virus–encoded small RNAs, *CISH* Chromogenic in situ hybridization

After 2 days of the HLH-94 regimen treatment, the patient’s high fever disappeared, and multiple laboratory tests gradually returned to normal (Table 1). One week after treatment, lymph node biopsy showed KFD (Fig. 1c). The patient was ultimately diagnosed with HLH-KFD. Whole-exome sequencing (WES) was used to detect HLH-related genes. WES results showed that mutations in the *TRAF3, C6,* and *RIPK1* genes were associated with immunity against infection but not with familial HLH [6].

To clarify the etiology, lymph node specimens were used to detect pathogenic microorganisms by mNGS. Through mNGS, human herpesvirus 7, human herpesvirus 4, and human herpesvirus 6 were revealed in lymph node biopsies. The patient was eventually diagnosed with virus-induced HLH-KFD. Hence, VP16 was quickly withdrawn from treatment regimens. Dexamethasone was gradually reduced and discontinued within 1 month. To date, he has remained HLH-KFD-free after a follow-up period of over one year.

Both HLH and KFD are rare diseases with complicated causes and diverse outcomes. HLH has been broadly categorized into primary and acquired forms. Primary HLH has autosomal recessive inheritance and incomplete penetrance with mutated genes, including *UNCI3D* and *STX11* [7]*.* Acquired HLH is usually associated with infection, tumor, rheumatism, or other underlying causes. Through a series of examinations, we excluded familial HLH, malignancy-associated HLH, and rheumatism associated HLH. The most common pathogen related to acquired HLH is EBV, especially in chronic active Epstein-Barr virus (CAEBV) [8]. Viruses can trigger not only HLH but also KFD [9]. Although EBV-related HLH is the most common type of viral-induced HLH in childhood, no evidence of EBV infection was found in our case. WES showed mutations in the *TRAF3*, *C6*, and *RIPK1* genes, which were not related to familial HLH [6]. mNGS is a new technology that has the potential to enhance our ability to track infectious diseases. Previous studies showed that mNGS in lung biopsy was valuable for the diagnosis of pathogenic microorganisms [10]. Through lymph node biopsy, our case was eventually diagnosed as HLH-KFD triggered by multiple human herpesviruses. Therefore, when the blood sample examinations for pathogens were negative, lymph node biopsy still gave us the opportunity to track pathogens by mNGS.

HLH treatment relies on the underlying cause and the severity of clinical presentation. Two key points for treatments are suppression of hyperinflammation and targeting of the underlying disease if possible [11]. Macrophage activation syndrome (MAS) constitutes one subtype of HLH that is induced by rheumatic disease. HLH-MAS was associated with a high mortality rate (22%–29%) [12, 13]. The HLH-94 treatment protocol, including VP16, was highly effective in treating hyperinflammation with HLH [14, 15], which reduced the mortality rate to 14% [16]. Patients with primary HLH and non-malignant serious HLH may be candidates for allogeneic hematopoietic stem cell transplantation, especially refractory/relapsing HLH, which may have genetic factors that are not currently known [14, 15]. On the other hand, appropriate antiviral treatment and immunomodulatory agents are usually effective for virus-induced non-EBV-HLH [9].

KFD is a self-limited inflammatory disease with unknown etiology that may be a viral or autoimmune disorder similar to systemic lupus erythematosus [4, 5]. Corticosteroids and/or symptomatic treatments are commonly used in clinical practice [5]. At present, there are few large samples of clinical research on HLH-KFD. The article with the largest number of patients reported 13 cases of HLH-KFD [17]. Clinicians may be confused by the following question: using VP16 immediately according to HLH treatment or using corticosteroids first according to KFD therapy?

To clarify this question, we conducted a literature review in the PubMed, Elsevier, Embase, BioRxiv, and MedRxiv databases. There were 57 cases of HLH-KFD (detailed information is shown in Supplementary Table 1). Four patients had been diagnosed with KFD prior to HLH. One patient developed KFD after the HLH diagnosis. The remaining patients were diagnosed with HLH and KFD during the same hospitalization.

The median age of HLH-KFD was 14 years, and adolescents were at high risk of HLH-KFD. Analyzing the initial treatment of HLH-KFD, we found that the mortality rate was 7.02% at initial treatment. Four deaths at initial treatment included two pregnant cases of HLH-KFD (Table 2). The prognosis of HLH-KFD with pregnancy was very poor. Two non-pregnant HLH-KFD patients died during initial treatment [18, 19], including one patient who died of multi-drug-resistant *Acinetobacter baumannii* bacteremia rather than the progression of HLH-KFD.

**Table 2:** Clinical characteristics and therapeutic effect of 57 HLH-KFD patients

Clinical features	Number of patients (%)
Gender, *n* (male/female)	31/26
Age, y, median (range)	14 (0.3–54)
Previous or present EBV infection, *n* (%)	20/57 (35.09%)
Mortality at initial treatment, *n* (%)	4/57 (7.02%)*
Early effective response to IVIG, steroids and/or other immunosuppressants, symptomatic treatment and/or antibiotic treatment, *n* (%)	49/57 (85.96%)
Treatment of VP16, *n* (%)	8/57(14.04%)
Effective response to VP16, *n* (valid/invalid)	7/1
HLH-KFD relapse, *n* (%)	6/53 (11.32%, excluding 4 deaths)

*Including two pregnant cases of HLH-KFD. One pregnant woman developed HLH-KFD at the 19th week of gestation; she was treated with HLH-94 protocol and died of liver failure. Another pregnant woman was admitted at the 29th week + 6 days of gestation with fever and abnormal blood tests; she was diagnosed with HLH on postpartum day 6. After the therapy of IVIG and acyclovir failed, she died on postpartum day 11 owing to disseminated intravascular coagulation and adult respiratory distress syndrome. Her autopsy showed Epstein-Barr virus RNA-induced HLH-KFD

*EBV* Epstein–Barr virus, *IVIG* intravenous immunoglobulin, *VP16* etoposide, *HLH-KFD* hemophagocytic lymphohistiocytosis associated with histiocytic necrotizing lymphadenitis

First-line treatment included symptomatic treatment and/or antibiotic treatment, intravenous immune globulin (IVIG), steroids and/or other immunosuppressants. Of 57 patients with HLH-KFD, eight patients improved quickly after anti-infective and symptomatic treatment, and the other patients were treated with IVIG, steroids and/or other immunosuppressants. The overall response rate was 85.96% with first-line treatment. Both HLH and KFD will induce abnormal immune and inflammatory conditions [4], and steroids and IVIG were common treatments. However, immunosuppressive therapy for HLH-MAS, including steroids and cyclosporin, caused a high mortality [12, 13]. Compared to HLH-MAS, HLH-KFD might have a good prognosis using IVIG, steroids and/or other immunosuppressants.

Patients failed from the first-line treatment were switched to VP16. A total of 7/8 patients responded to VP16. In the 53 patients (excluding four mortalities) who responded to treatments, six cases of HLH relapsed later. The HLH-94 protocol, including VP16, was highly effective and reduced the HLH-MAS mortality rate to 14% [14, 15]. For HLH-KFD patients who had failed the first-line treatment, the HLH-94 protocol was also effective.

Based on the literature review, less than half of the patients were able to identify the pathogenic factors (Supplementary Table 2). Infections and autoimmune disorders were the leading predisposing factors of HLH-KFD. Hematologic neoplasms and mutations of familial HLH genes could also cause HLH-KFD. In infection-associated HLH-KFD, various viruses are the main causes. Due to the diversity of viruses and the difficulty of diagnosis, mNGS should be an important tool to exploit the cause of HLH-KFD. Previous or present EBV infections in 35.09% of patients suggested the vital role of EBV in the occurrence of HLH-KFD.

Seven of eight patients who did not respond to first-line treatment remained responsive to treatments including VP16. Five patients switched to a regimen containing VP16 because of disease progression, such as epileptic seizure, capillary leakage syndrome, hypoxemia, respiratory failure and marked hemophagocytosis in cerebrospinal fluid (CSF). One patient switched to a regimen containing VP16 after the onset of disseminated intravascular coagulation and then died. The key pathogenesis of HLH was cytokine storm caused by abnormal activation of macrophages. Capillary leakage syndrome, hypoxemia, and respiratory failure were signs of cytokine storm progression, which called the timely adjustment in treatments. Patients with central nervous system symptoms and/or abnormal CSF at diagnosis had significantly increased mortality [12]. A previous study suggested that the HLH-MAS mortality rate was associated with shock, thrombocytopenia and B cell lymphoma [20], which demonstrated that VP16 was essential to save patient life in the conditions described above. For our case, once the diagnosis of virus-induced HLH-KFD was confirmed, VP16 was quickly withdrawn from treatment. Dexamethasone was gradually reduced and discontinued within a month.

In summary, HLH-KFD was mainly induced by infections and autoimmune factors, of which adolescents were at high risk. Of note, steroids and IVIG should be used as the first line treatment, followed by VP16 as soon as possible for patients who are unresponsive to steroids. To the best of our knowledge, this might be the first detailed review about the clinical profile, etiology, and outcome of HLH-KFD, warranting further clinical investigation.

## Supplementary Information

Below is the link to the electronic supplementary material.

Supplementary file1 (DOCX 105 KB)

## Data Availability

The raw data supporting the conclusions of this article will be made available by the authors, without undue reservation. All data obtained and analyzed in this study were available from the corresponding author in a reasonable request.
