# Supplementary material for: Clinical profile, etiology, and outcome of hemophagocytic lymphohistiocytosis associated with histiocytic necrotizing lymphadenitis
Source: World J Pediatr. 2023 Jan 3;19(4):406–10. doi: 10.1007/s12519-022-00660-z (PMC10060272; doi:10.1007/s12519-022-00660-z)
Supplement: Supplementary file 1 — Supplementary file1 (DOCX 105 KB) [file 12519_2022_660_MOESM1_ESM.docx]

**Supplemental Information**

**Contents**

**Supplemental Table 1.**

**Supplemental Table 2.**

**Supplemental Table 1.** Basic features, etiology, treatment and prognosis of 57 HLH-KFD cases

| Case no. | Age  (years) | Gender | Autoimmune  testing | HLH related gene | Treatment | Reason for VP16 | Outcome | Etiology or predisposing factors |
| --- | --- | --- | --- | --- | --- | --- | --- | --- |
| 1^[1]^ | 6 | F | NEG | Homozygous mutations in RAB27A | High dose CSI with MTX firstly, followed by HLH-04 protocol and alemtuzumab | First-line treatment failure and central nervous system involvement | Improvement | Uveitis and dengue |
| 2^[2]^ | 26 | M | SLE | NA | Oral PRED (40mg/d) along with HCQ firstly. Treatment failure and diagnosis of HLH followed by increased PRED (80mg/d) |  | Improvement | SLE |
| 3^[3]^ | 35 | F | NEG | NA | MEP (1g/d) for 3 days, followed by DXM (20 mg/d) and VP16 (75 mg/m^2^, twice a week). | First-line treatment failure | Spontaneous abortion next day, died two weeks after treatment with VP16 | Upper respiratory infection 6 weeks before onset (at 13 weeks of gestation) |
| 4^[4]^ | 1.75 | M | NA | NEG | PRED (2 mg/kg) |  | Improvement | JMML (KRAS mutation (n38(G > A))) |
| 5^[5]^ | 6 | M | NEG | NA | PRED based on abscess excision and antibiotic. Treatment failure, followed by DXM, CYCL, VP16 | NA | Improvement | Periodontal abscess. KFD 2 years before HLH |
| 6^[6]^ | 1 | F | NA | NA | CYCL and oral PRED |  | Improvement | JIA for 1 year. KFD before HLH |
| 7^[7]^ | 13 | F | NEG | NA | IVIG +MEP (1g/d for 3 days) firstly followed by DXM+VP16 | First-line treatment failure and respiratory failure | Improvement | No predisposing factor found |
| 8^[8]^ | 14 | M | NEG | NA | IVIG (1g/kg/d x 2 days) +oral PRED (2 mg/kg/d) |  | Improvement | Prior infection with EBV, EBER-1 negative in lymph node biopsy |
| 9^[8]^ | 10 | F | NEG | NA | IVIG (1g/kg/d x 2 days) +oral pred (2mg/kg/d) |  | Improvement | No predisposing factor found |
| 10^[9]^ | 15 | F | NA | NA | PRED (60mg/day) |  | Improvement | Parvovirus B19 infection, PV-B19 DNA positive in histopathology |
| 11^[10]^ | 0.3 | M | NA | NA | Antibiotic + symptomatic treatment |  | Improvement | No predisposing factor found |
| 12^[11]^ | 54 | F | NEG | NA | Antibiotic + symptomatic treatment |  | Improvement | No predisposing factor found |
| 13^[12]^ | 50 | M | SLE | NA | CSI |  | Improvement, died of multidrug resistant Acinetobacter baumanii bacteremia later | SLE |
| 14^[13]^ | 8.9 | M | NEG | NA | IVIG +MEP |  | Improvement | Previous EBV infection |
| 15^[13]^ | 2.1 | M | POS (ANA) | NA | IVIG +MEP |  | Improvement | Previous EBV infection |
| 16^[13]^ | 5.3 | M | NEG | NA | IVIG +MEP |  | Improvement | No predisposing factor found |
| 17^[13]^ | 13.9 | M | NEG | NA | MEP firstly. treatment failure, followed by DXM, CYCL, VP16, CTX | NA | Improvement, HLH relapsed 9 months later | Previous EBV infection |
| 18^[13]^ | 9.9 | F | NA | NA | Symptomatic treatment |  | Improvement | No predisposing factor found |
| 19^[13]^ | 11.6 | F | NA | NA | IVIG |  | Improvement | Previous EBV infection |
| 20^[13]^ | 14 | M | POS (ANA) | NA | Symptomatic treatment |  | Improvement | Previous EBV infection |
| 21^[13]^ | 3.8 | M | NEG | NA | IVIG +MEP |  | Improvement | Previous EBV infection |
| 22^[13]^ | 5.3 | M | NEG | NA | CSI, CYCL |  | Improvement | Previous EBV infection |
| 23^[13]^ | 6 | F | NA | NA | Symptomatic treatment |  | Improvement，SLE and sicca syndrome later | Previous EBV infection |
| 24^[13]^ | 8.3 | M | POS (ANA) | NA | CSI |  | Improvement | Previous EBV infection |
| 25^[13]^ | 12.7 | M | POS (ANA) | NA | IVIG +MEP |  | Improvement | Previous EBV infection |
| 26^[13]^ | 3 | F | POS (ANA) | NA | MEP + ruxolitinib |  | Improvement | Previous EBV infection |
| 27^[14-16]^ | 12 | M | NA | NA | CSI |  | Improvement, HLH relapse 2 years later | No predisposing factor found |
| 28^[14-16]^ | 14 | M | NA | NA | IVIG, acyclovir, DXM, VP16 | NA | Improvement, HLH relapse 7 years later | EBV infection |
| 29^[14-16]^ | 5 | F | NA | NA | PRED, VP16, DXM | NA | Died | No predisposing factor found |
| 30^[14-16]^ | 14 | F | NA | NA | IVIG, DXM, VP16, CYCL | NA | Improvement | No predisposing factor found |
| 31^[14-16]^ | 8 | M | NA | NA | PRED |  | Improvement | EBV infection |
| 32^[17]^ | 19 | M | NA | No genetic abnormalities related to HLH by NGS | DXM (10mg/m^2^/day) |  | Improvement | KFD 3 years before HLH with treatment of HCQ |
| 33^[15, 16, 18]^ | 40 | M | NEG | NA | Symptomatic treatment(naproxen) |  | Improvement | No predisposing factor found |
| 34^[15]^ | 4.6 | M | NEG | NA | CSI+IVIG followed by HLH-04 protocol 2 weeks later | First-line treatment failure and capillary leakage syndrome | Improvement | No predisposing factor found |
| 35^[19]^ | 21 | M | NEG | NA | PRED |  | Improvement | Sweet's syndrome before, RIG-I expression indicates infection despite virus-negative serology (EBV, herpes simplex virus, CMV and HHV-7) |
| 36^[20, 21]^ | 24 | F | NEG | NA | Fever at 29 weeks + 6 days gestation, diagnosis of HLH on postpartum day 6, treated by IVIG |  | Died of DIC and adult respiratory distress syndrome | Diagnosis of HLH on postpartum day 6, autopsy showed EBV RNA-induced HLH-KFD |
| 37^[15, 22]^ | 36 | F | NA | NA | DXM |  | Improvement, HLH recurrence 3 months later because of coronavirus | Positive of EBV and parvovirus B19 by PCR |
| 38^[23]^ | 21 | M | NA | NA | PRED + anti-lymphoma therapy (pralatrexate) |  | Improvement | SPTL |
| 39^[15, 16]^ | 30 | M | NA | NA | PRED |  | Improvement | Upper respiratory tract infection, and previous EBV infection |
| 40^[16, 24]^ | 17 | F | NA | NA | MEP +IVIG |  | Improvement | Highly suspected virus infection |
| 41^[16, 25]^ | 15 | F | NA | NA | PRED |  | Improvement | No predisposing factor found |
| 42^[16, 26, 27]^ | 37 | F | SLE | NA | CSI |  | Improvement | SLE |
| 43^[8, 16, 21]^ | 10 | F | POS (ANA, C3, C4) | NA | IVIG +MEP |  | Improvement | Previous EBV infection |
| 44^[8, 16, 21]^ | 14 | M | POS (C3, C4) | NA | IVIG +MEP |  | Improvement | Previous EBV infection |
| 45^[16, 28]^ | 17 | F | NA | NA | MEP |  | Improvement | No predisposing factor found |
| 46^[21]^ | 11 | M | NA | NA | PRED |  | Improvement, HLH relapse later | No predisposing factor found |
| 47^[21]^ | 13 | M | No | No alteration in the perforin or Munc13-4 genes. | PRED |  | Improvement, HLH relapse 26 months later | No predisposing factor found |
| 48^[26]^ | 16 | M | NA | NA | Symptomatic treatment |  | Improvement | Recent EBV infection detected by serology tests (anti-EBV capsid IgM+) |
| 49^[29]^ | 17 | F | NA | NA | MEP, IVIG, acyclovir |  | Improvement | No predisposing factor found |
| 50^[30]^ | 17 | M | SLE | NA | IVIG +MEP |  | Improvement | SLE |
| 51^[31]^ | 16 | F | NA | No perforin gene mutations | CYCL, MEP |  | Improvement | No predisposing factor found |
| 52^[32]^ | 30 | M | NA | NA | MEP |  | Improvement | No predisposing factor found |
| 53^[33]^ | 49 | F | NA | NA | High dose CSI for 3 days, tapered off |  | Improvement | Abscess of staphylococcus epidermidis before 1 week |
| 54^[34]^ | 13 | F | NA | NA | CSI |  | Improvement | No predisposing factor found |
| 55^[35]^ | 16 | F | NA | NA | Symptomatic treatment |  | Improvement | Elevation of IgM of dengue fever antibody |
| 56^[36]^ | 1.6 | M | NA | NA | CSI |  | Improvement | No predisposing factor found |
| 57^[37]^ | 15 | F | NA | Perforin gene Ala91Val mutation | MEP (2mg/kg/day) and CYCL (2mg/kg) |  | Improvement | sJIA |

CSI, corticosteroids; MTX, methotrexate; DXM, dexamethasone; CYCL, cyclosporine; VP16, etoposide; PRED, prednisolone; HCQ, hydroxychloroquine; MEP, Methylprednisolone; IVIG, Intravenous immunoglobulin; CTX, cyclophosphamide; NEG, negative; POS, positive; EBV, Epstein-Barr virus; PV-B19, Parvovirus B19 infection; sJIA, systemic juvenile idiopathic arthritis; SLE, systemic lupus erythematosus; HLH, hemophagocytic lymphohistiocytosis; KFD, histiocytic necrotizing lymphadenitis; HLH-KFD, hemophagocytic lymphohistiocytosis associated with histiocytic necrotizing lymphadenitis; SPTL, subcutaneous panniculitis-like t-cell lymphoma; JMML, juvenile myelomonocytic leukemia.

**Supplemental Table 2.** Etiology of 57 HLH-KFD patients

| **Etiology** | **No. of patients (*n*)** |
| --- | --- |
| - **Definite etiology** | 23/57 |
| - **Single factor** | 20 |
| **Infection** | 12 |
| Bacteria abscess | 2 |
| EBV | 4 (including one pregnant woman) |
| Parvovirus B19 | 1 |
| Parvovirus B19 and EBV | 1 |
| Dengue fever virus | 1 |
| Upper respiratory virus | 3 (including one pregnant woman) |
| **Rheumatologic diseases** | 6 |
| sJIA | 1 |
| SLE | 4 |
| SLE and sicca syndrome | 1 |
| **Cancer** | 2 |
| JMML | 1 |
| SPTL | 1 |
| - **Complex factor** | 3 |
| Autoimmune uveitis and dengue fever with homozygous mutations in RAB27A | 1 |
| sJIA with Ala91Val mutation | 1 |
| Sweet’s syndrome and infection | 1 |
| - **Unclear etiology** | 34/57 |
| Abnormality autoimmune texts with previous EBV infection, without other definite causes | 7 |
| Previous EBV infection without other definite causes | 6 |
| No predisposing factor found | 21 |

EBV, Epstein-Barr virus; VP16, etoposide; IVIG, Intravenous immunoglobulin; sJIA, systemic juvenile idiopathic arthritis; SLE, systemic lupus erythematosus; HLH-KFD, hemophagocytic lymphohistiocytosis associated with histiocytic necrotizing lymphadenitis; SPTL, subcutaneous panniculitis-like t-cell lymphoma; JMML, juvenile myelomonocytic leukemia.

**References**

1 Listernick R. A 6-year-old girl with 'pink eye' for several months. Pediatr Ann 2010;39:267-70.

2 Pradhan S, Sirka CS, Dash G, Sahu K, Rout AN. A challenging case of Kikuchi-Fujimoto disease with systemic lupus erythematosus complicated with hemophagocytic lymphohistiocytosis in a young man. Indian J Dermatol Venereol Leprol 2021;87:78-82.

3 Giard JM, Decker KA, Lai JC, Gill RM, Logan AC, Fix OK. Acute Liver Failure Secondary to Hemophagocytic Lymphohistiocytosis during Pregnancy. ACG Case Rep J 2016;3:e162.

4 Gerritsen A, Lam K, Marion Schneider E, van den Heuvel-Eibrink MM. An exclusive case of juvenile myelomonocytic leukemia in association with Kikuchi's disease and hemophagocytic lymphohistiocytosis and a review of the literature. Leuk Res 2006;30:1299-303.

5 Mahadeva U, Allport T, Bain B, Chan WK. Haemophagocytic syndrome and histiocytic necrotising lymphadenitis (Kikuchi's disease). J Clin Pathol 2000;53:636-8.

6 Ramanan AV, Wynn RF, Kelsey A, Baildam EM. Systemic juvenile idiopathic arthritis, Kikuchi's disease and haemophagocytic lymphohistiocytosis--is there a link? Case report and literature review. Rheumatology (Oxford) 2003;42:596-8.

7 Kim YM, Lee YJ, Nam SO, Park SE, Kim JY, Lee EY. Hemophagocytic syndrome associated with Kikuchi's disease. J Korean Med Sci 2003;18:592-4.

8 Chen JS, Chang KC, Cheng CN, Tsai WH, Su IJ. Childhood hemophagocytic syndrome associated with Kikuchi's disease. Haematologica 2000;85:998-1000.

9 Yufu Y, Matsumoto M, Miyamura T, Nishimura J, Nawata H, Ohshima K. Parvovirus B19-associated haemophagocytic syndrome with lymphadenopathy resembling histiocytic necrotizing lymphadenitis (Kikuchi's disease). Br J Haematol 1997;96:868-71.

10 Kim HA, Im SA, Chung NG, Kang JH, Park GS. Disseminated Kikuchi disease associated with hemophagocytic syndrome in an infant: whole-body MRI. Indian J Pediatr 2011;78:616-9.

11 Hoogstins HA, Kibbelaar RE, Ubels FL, Hemmelder MH, Hoogendoorn M. Expanding the clinical spectrum of self-limiting, rare Kikuchi disease - A case with overwhelming multi-organ involvement. Neth J Med 2017;75:112-6.

12 Kampitak T. Fatal Kikuchi-Fujimoto disease associated with SLE and hemophagocytic syndrome: a case report. Clin Rheumatol 2008;27:1073-5.

13 Yang Y, Lian H, Ma H, Zhao Y, Zhang Q, Zhang L, et al. Hemophagocytic Lymphohistiocytosis Associated with Histiocytic Necrotizing Lymphadenitis: A Clinical Study of 13 Children and Literature Review. J Pediatr 2021;229:267-74.e263.

14 Lim GY, Cho B, Chung NG. Hemophagocytic lymphohistiocytosis preceded by Kikuchi disease in children. Pediatr Radiol 2008;38:756-61.

15 Duan W, Xiao ZH, Yang LG, Luo HY. Kikuchi's disease with hemophagocytic lymphohistiocytosis: A case report and literature review. Medicine (Baltimore) 2020;99:e23500.

16 Nishiwaki M, Hagiya H, Kamiya T. Kikuchi-Fujimoto Disease Complicated with Reactive Hemophagocytic Lymphohistiocytosis. Acta Med Okayama 2016;70:383-8.

17 Lee SM, Lim YT, Jang KM, Gu MJ, Lee JH, Lee JM. Hemophagocytic lymphohistiocytosis with recurrent Kikuchi-Fujimoto disease. Yeungnam Univ J Med 2021;38:245-50.

18 Khan FY, Morad NA, Fawzy Z. Kikuchi's disease associated with hemophagocytosis. Chang Gung Med J 2007;30:370-3.

19 Koga T, Takano K, Horai Y, Yamasaki S, Nakamura H, Mizokami A, et al. Sweet's syndrome complicated by Kikuchi's disease and hemophagocytic syndrome which presented with retinoic acid-inducible gene-I in both the skin epidermal basal layer and the cervical lymph nodes. Intern Med 2013;52:1839-43.

20 Chmait RH, Meimin DL, Koo CH, Huffaker J. Hemophagocytic syndrome in pregnancy. Obstet Gynecol 2000;95:1022-4.

21 Lin YW, Horiuchi H, Ueda I, Nambu M. Recurrent hemophagocytic lymphohistiocytosis accompanied by Kikuchi's disease. Leuk Lymphoma 2007;48:2447-51.

22 Gowarty J, Oda J, Cable C. Hemophagocytic lymphohistiocytosis. Proc (Bayl Univ Med Cent) 2018;31:350-1.

23 Notaro E, Shustov A, Chen X, Shinohara MM. Kikuchi-Fujimoto Disease Associated With Subcutaneous Panniculitis-Like T-Cell Lymphoma. Am J Dermatopathol 2016;38:e77-80.

24 Okuda T, Yumoto Y. [Subacute necrotizing lymphadenitis with a clinical course mimicking virus-associated hemophagocytic syndrome]. Rinsho Ketsueki 1994;35:689-93.

25 Watanabe S, Mochizuki H, Nakashima I, Itoyama Y. [A case of primary Sjögren's syndrome with CNS disease mimicking chronic progressive multiple sclerosis]. Rinsho Shinkeigaku 1998;38:658-62.

26 Lee HY, Huang YC, Lin TY, Huang JL, Yang CP, Hsueh T, et al. Primary Epstein-Barr virus infection associated with Kikuchi's disease and hemophagocytic lymphohistiocytosis: a case report and review of the literature. J Microbiol Immunol Infect 2010;43:253-7.

27 Wano Y, Ebata K, Masaki Y, Takeshita S, Ogawa N, Kim CG, et al. [Histiocytic necrotizing lymphadenitis (Kikuchi-Fujimoto's disease) accompanied by hemophagocytosis and salivary gland swelling in a patient with systemic lupus erythematosus]. Rinsho Ketsueki 2000;41:54-60.

28 Kelly J, Kelleher K, Khan MK, Rassam SM. A case of haemophagocytic syndrome and Kikuchi-Fujimoto disease occurring concurrently in a 17-year-old female. Int J Clin Pract 2000;54:547-9.

29 Lin HC, Su CY, Huang SC. Kikuchi's disease in Asian children. Pediatrics 2005;115:e92-6.

30 Vithoosan S, Karunarathna T, Shanjeeban P, Piranavan P, Matthias T, Gamlaksha D, et al. Kikuchi-Fujimoto disease associated with systemic lupus erythematosus complicated with hemophagocytic lymphohistiocytosis: a case report. J Med Case Rep 2019;13:173.

31 Lelii M, Senatore L, Amodeo I, Pinzani R, Torretta S, Fiori S, et al. Kikuchi-Fujimoto disease in children: two case reports and a review of the literature. Ital J Pediatr 2018;44:83.

32 Dumas G, Prendki V, Haroche J, Amoura Z, Cacoub P, Galicier L, et al. Kikuchi-Fujimoto disease: retrospective study of 91 cases and review of the literature. Medicine (Baltimore) 2014;93:372-82.

33 Cao NT, Nguyen HT, Pham VHT, Doan TTP, Le LB, Bui KD, et al. Reactive Hemophagocytic Lymphohistiocytosis-Associated Kikuchi-Fujimoto Disease After a Staphylococcus epidermidis Cutaneous Infection: The First Case Report. J Clin Rheumatol 2021;27:e96-7.

34 Yoo IH, Na H, Bae EY, Han SB, Lee SY, Jeong DC, et al. Recurrent lymphadenopathy in children with Kikuchi-Fujimoto disease. Eur J Pediatr 2014;173:1193-9.

35 Sykes JA, Badizadegan K, Gordon P, Sokol D, Escoto M, Ten I, et al. Simultaneous Acquired Self-limited Hemophagocytic Lymphohistiocytosis and Kikuchi Necrotizing Lymphadenitis in a 16-Year-Old Teenage Girl: A Case Report and Review of the Literature. Pediatr Emerg Care 2016;32:792-8.

36 Inamo Y. The Difficulty of Diagnosing Kikuchi-Fujimoto Disease in Infants and Children Under Six Years Old: Case Report and Literature Review. Cureus 2020;12:e7383.

37 Marsili M, Nozzi M, Onofrillo D, Sieni E, Chiarelli F, Breda L. Kikuchi disease, macrophage activation syndrome, and systemic juvenile arthritis: a new case associated with a mutation in the perforin gene. Scand J Rheumatol 2015;44:429-30.
